# Using a Virtual Serious Game (Deusto-e-motion1.0) to Assess the Theory of Mind in Primary School Children: Observational Descriptive Study

**DOI:** 10.2196/12971

**Published:** 2020-04-02

**Authors:** Esther Lázaro, Imanol Amayra, Juan Francisco López-Paz, Oscar Martínez, Manuel Pérez Alvarez, Sarah Berrocoso, Mohammad Al-Rashaida, Maitane García, Paula Luna, Paula Pérez-Núñez, Alicia Aurora Rodriguez, Paula Fernández, Pamela Parada Fernández, Mireia Oliva-Macías

**Affiliations:** 1 University of Deusto Bilbao Spain

**Keywords:** serious game, theory of mind, facial emotion recognition, children

## Abstract

**Background:**

Given the interactive media characteristics and intrinsically motivating appeal, virtual serious games are often praised for their potential for assessment and treatment.

**Objective:**

This study aims to validate and develop normative data for a virtual serious game (Deusto-e-motion1.0) for the evaluation of emotional facial expression recognition and social skills, both of which are components of the theory of mind.

**Methods:**

A total of 1236 children took part in the study. The children were classified by age (8-12 years old), gender (males=639, females=597), and educational level (between the third and sixth years of Primary Education). A total of 10 schools from the Basque Country and 20 trained evaluators participated in this study.

**Results:**

Differences were found in Deusto-e-motion1.0 scores between groups of children depending on age and gender. Moreover, there was a moderately significant correlation between the emotional recognition scores of Deusto-e-motion1.0 and those of the Feel facial recognition test.

**Conclusions:**

Deusto-e-motion1.0 shows concurrent validity with instruments that assess emotional recognition. Results support the adequacy of Deusto-e-motion1.0 in assessing components of the theory of mind in children.

## Introduction

Serious games represent a growing area of computer applications used to improve or evaluate different skills. They are appealing, interactive, enhance ecological validity, and allow players to take on realistic roles to cope with problems and to make decisions [1,2]. Games are entertaining, but they can also be educational [3].

The use of computer software has several advantages: the environment is predictable, consistent, free from social demands, and users can work alone. Furthermore, lessons can be repeated, and motivation can be maintained through rewards and feedback [4]. Virtual and mixed realities present the possibility of creating new, immersive, and motivational places where patients can be evaluated and trained while playing [5].

There are various serious games available, such as some for training skills [6], for prevention [7], for psychological therapy [8], or for cognitive training. Other types of games help users to deal with those with special needs, such as the elderly [9], people with physical disabilities, or blind children [10]. An example of a serious game is Happy Farm [11], a software for young people designed to increase their awareness of the risks related to psychoactive substances. Another program, VEPSY (updated telemedicine and portable virtual environments for clinical psychology) [3], was created to investigate the effects of virtual reality systems aimed at dealing with several clinical disorders, such as social phobia, obesity, bulimia, or male impotence. The project combines treatments and assessments with virtual reality. Similar games have been developed to induce mood enhancement on both clinical and nonclinical samples [12]. The EMMA project (Engaging Media for Mental Health Applications) provides innovative ways of coping with distressful emotions for users who suffer psychological problems, users with restricted mobility, or the general population [13]. Another group of serious games has been created to assess and train the components of the theory of mind.

The theory of mind covers mental skills related to understanding, explaining, and predicting the psychological states of oneself and others [14]. The theory of mind was first established in animal studies with chimpanzees [15] and later in infant developmental psychology and autism [16]. The theory of mind permits typically functioning individuals to infer the mental and emotional states of others as a means of engaging reciprocal communication and maintaining relationships. Recognition of emotional facial expressions is an essential part of the theory of mind. The face is the way that emotions can be exteriorized and expressed in a nonverbal way, something essential for a person to adapt to the social environment around them, as shown in “Mind Reading: The Interactive Guide to Emotions” (Jessica Kingsley Publishers, London, United Kingdom). This is a multimedia computer program that has been used to address emotion recognition.

Attempts to teach components of the theory of mind to people with autism spectrum conditions have used computer-based training [17-19] or virtual environments [20]. Golan and Baron Cohen used “Mind Reading: The Interactive Guide to Emotions,” during a study on adults with Asperger syndrome or high-functioning autism [21]. They used it as an interactive guide to teach emotions in a systematic and comprehensive format, as it includes an emotion library, a learning center, and a game zone. The results of this study revealed that the use of the program significantly improves emotional recognition skills in adults with autistic spectrum conditions.

In 2002, Bölte [18] developed a computer-based program to teach and test the ability to identify facial emotions, known as the “Frankfurt Test and Training of Social Affect” (FEFA). The training was conducted for five weeks, for two hours a week, and the participants improved significantly on the facial recognition task. The Motion Picture Mind Reading test is a naturalistic mind-reading test designed to measure individual differences among a young adult population watching TV films showing characters in various social situations [22]. There are several collections of material and databases with facial emotion information and photographs, pictures [23,24], or virtual faces [25]. Different questionnaires have been developed to assess facial recognition ability, including the Florida Affect Battery test [26] or the Feel test [27]. However, a few types of software evaluate facial emotion recognition and empathy in children through serious virtual games.

The present study evaluated a new program, Deusto-e-motion1.0, which was developed to assess and train components of the theory of mind in children between the ages of 8-11 years old. Specifically, this paper presents the development and preliminary evaluation of the Spanish version of Deusto-e-motion1.0 to test the recognition of facial emotions in a sample of 1236 children.

## Methods

### Participants

A sample of children between the ages of 8-11 years old was chosen. The recognition of emotional facial expression improves between the ages of 8-14 years old, a period in which maturation processes associated with brain development occur [28]. The total sample was composed of 1236 children (males=639, females=597). The mean age was 9.58 (SD 1.11), with a breakdown of 269 8 year olds (males=148, females=121), 332 9 year olds (males=169, females=163), 290 10 year olds (males=151, females=139), and 345 11 year olds (males=171, females=174). Participants were excluded if there was any indication of an existing neurological or psychiatric disorder, according to the school psychologist’s criteria. The inclusion criteria were: speaking Spanish, being between 8-11 years old, between the third and sixth years of Primary Education, and having a normal IQ range (>90). For the fulfillment of the inclusion criterion of the IQ, the opinion of the team of professors of the schools in which the study was carried out was considered. Signed parental or school consent was obtained from all participants before beginning the study, and no remuneration was provided to either the students or their parents for taking part.

### Instruments

#### E-motion1.0

This program contains two sections and requires about 20 minutes to be completed (Tables 1 and 2). It is designed to be played on a personal computer during a psychosocial skills assessment. Each level follows a preset structure that integrates static and virtual scenes. Its target audience is 8-11 year-old males and females. Deusto-e-motion1.0 has two versions: (1) a virtual version which includes a head-mounted display, a motion tracker, and a joystick input device; and (2) a serious game version. The present study presents the validation results of the serious game’s Spanish version of Deusto-e-motion1.0.

**Table 1:** Summary of section 1 of Deusto-e-motion1.0.

Section	Emotions	Type of variable
Static faces	Neutral Happiness Anger Sadness Fear Surprise Disgust	Type of emotion chosen (nominal) Correct/incorrect (nominal) Reaction time (continuous)
Dynamic faces I	Happiness Anger Sadness Fear Surprise Disgust	Type of emotion chosen (nominal) Correct/incorrect (nominal) Reaction time (continuous)
Dynamic faces II	Neutral Happiness Anger Sadness	Type of emotion chosen (nominal) Correct/incorrect (nominal) Reaction time (continuous)
Static faces II	Neutral Happiness Anger Sadness Fear Surprise Disgust	Type of emotion chosen (nominal) Correct/incorrect (nominal) Reaction time (continuous)

**Table 2:** Summary of section 2 of Deusto-e-motion1.0.

Section	Items	Type of variable
Virtual scenes I	Three static scenes How would you feel about it? How would he/she feel?	Type of emotion chosen (nominal) Reaction time (continuous)
Virtual scenes II	Fourteen virtual scenes How would you feel about it? How would he/she feel?	Type of emotion chosen (nominal) Reaction time (continuous)

This instrument was developed by a team of multidisciplinary professionals: psychologists, psychopedagogues, and computer scientists. For the development of the first section of the instrument, visual stimuli were designed with virtual reality tools. Baron Cohen's facial emotional expression criteria were used, as found in “Mind Reading: The Interactive Guide to Emotions.” Virtual stimuli were chosen because of the higher possibility of controlling expression features. This section was validated with a pilot test with students of psychology and children between the ages of 8-12 years old. Depending on the results obtained, the details of the emotional expressions were modified to obtain a percentage of agreement between emotion and facial expression of at least 90%. The expression of fear was one of the most complicated to carry out because it could be confused with the expression of surprise.

**Figure 1:**
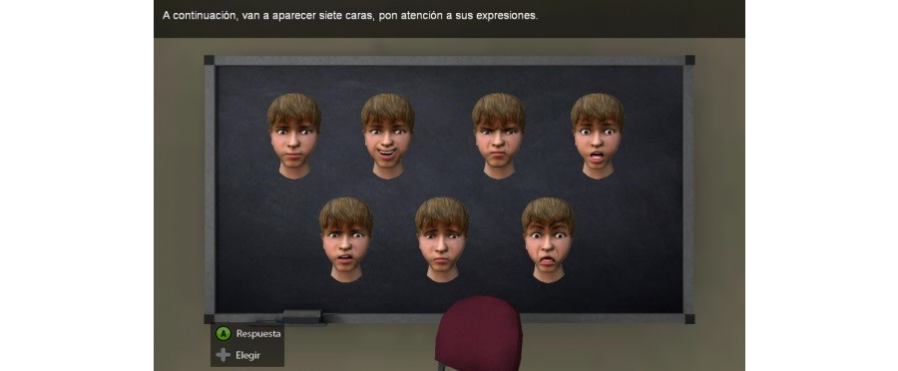
First section of Deusto-e-motion1.0: facial recognition.

The first section of Deusto-e-motion1.0 measures the ability to recognize facially-expressed basic emotions (Figure 1). The internationally known and applied cross-cultural concept of six basic emotions (happiness, sadness, anger, disgust, fear, surprise, and neutral), proposed by Ekman [29], was the reference for the selection of the pictures. This first block consists of four sections of 24 items: (1) seven static facial emotions; (2) six dynamic facial emotions, which include faces changing from neutral to another emotion; (3) four dynamic facial emotions which show faces changing from one emotion to another; and (4) seven static facial emotions. There are two blocks with static facial emotions to control the learning effect. Each face is presented on a computer screen for a maximum 30 seconds. Subjects must classify the respective emotion by clicking on the appropriate label in a forced-choice format (happiness, sadness, anger, disgust, fear, surprise, or neutral). Responses for all tasks are scored as correct or incorrect. The Deusto-e-motion1.0 automatically records the sum of total correct answers, the sum of static facial emotions scores, the sum of dynamic facial emotions scores, the error scores, and the reaction time for each emotion.

**Figure 2:**
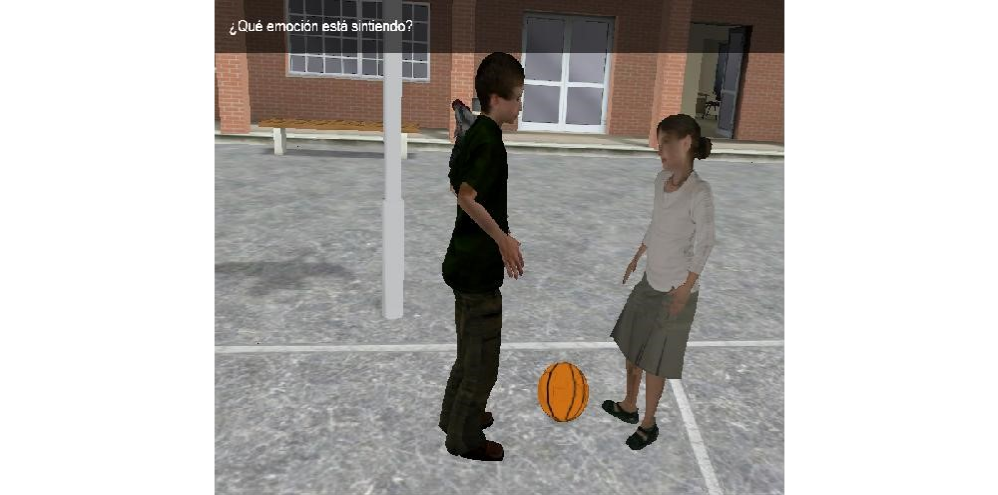
Second section of Deusto-e-motion1.0, virtual scenes at school: social situation in relation to choice in-game.

**Figure 3:**
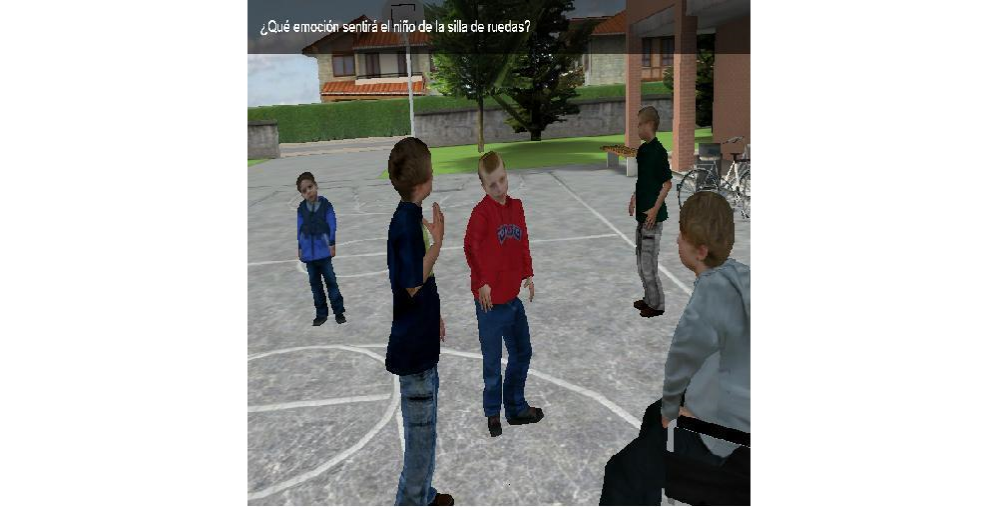
Second section of Deusto-e-motion1.0, virtual scenes at school: social situation with a boy in a wheelchair.

**Figure 4:**
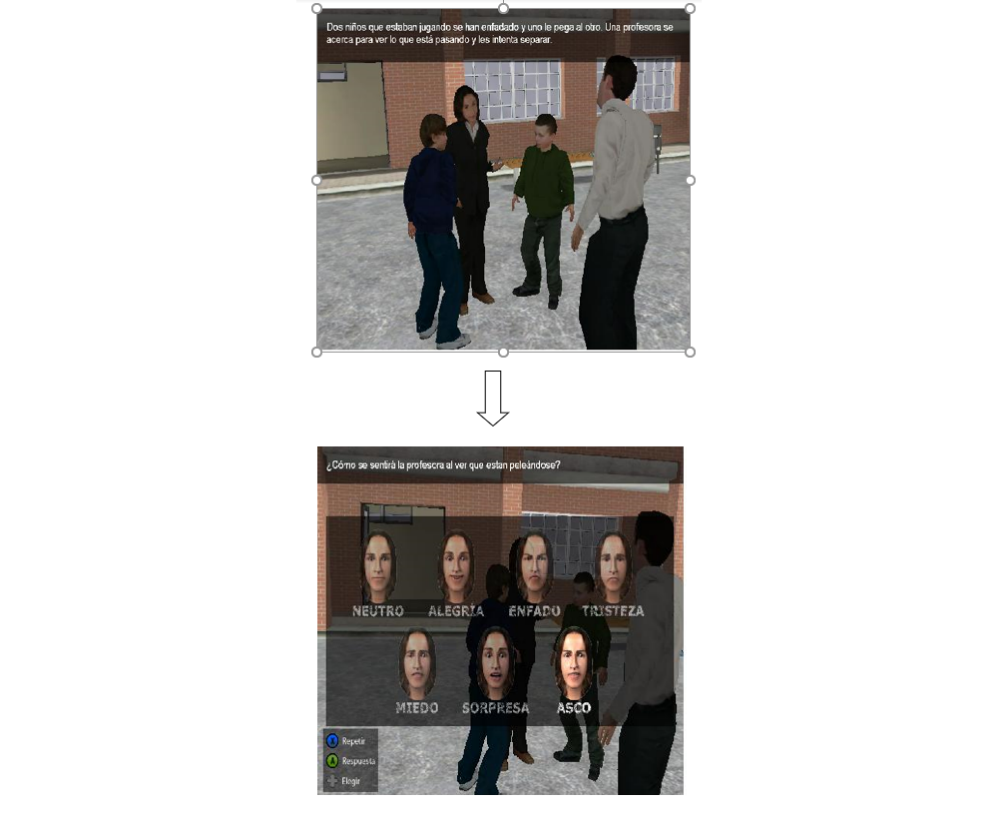
Second section of Deusto-e-motion1.0, virtual scenes at school: social situation with students and teachers, and answer options.

The second section consists of different virtual scenes placed in a virtual school setting (Figures 2 and 3). Figure 4 shows scene and answer options. This section was developed based on potential situations which may occur in the daily lives of children aged between 8-12 years old. For this reason, the school context was chosen, with a focus on the school playground. Social situations are presented that are related to problems or conflicts that can evoke emotions in other people as well as in oneself. The narrative develops through 30 items, each one lasting about half a minute or a full minute. After presenting each situation, the participant is asked to choose among the six emotions (happiness, sadness, anger, disgust, fear, surprise, and neutral). An example of a situation would be:

Your friends have planned an unexpected party for your birthday. How would you feel? How would they feel?

In addition to registering the choice of answers, Deusto-e-motion1.0 recorded the time taken by participants to select and answer each question. This test has had a previous validation study [30].

#### Feel

Feel is a computer-based test that measures the ability to recognize facially-expressed basic emotions [27]. This test was used together with Deusto-e-motion1.0 to obtain concurrent validity ratings. It consists of 42 photographs showing facial displays of six basic emotions (anger, fear, sadness, happiness, surprise, and disgust), developed by Matsumoto and Ekman [23], which are presented on a computer screen. Subjects must quickly and accurately classify the respective emotion by clicking on the appropriate label in a forced-choice format. In total, 42 pictures of adults are shown, and seven examples of each six emotions are used. The Feel test score takes the correct answers, error scores, and reaction time for all emotions. The test shows the highest reliability of this form of assessment (Cronbach alpha=.77). Over the years, a database with over 600 healthy subjects has been collected [27]. This test previously had a Spanish validation study, including a sample consisting of a total of 1189 school children aged between 8-11 years old, with 594 boys and 594 girls, and with a Cronbach alpha of 0.82 [31].

### Procedure

The validation test took eight months to be completed. Children were recruited from ten different schools in the Basque Country (Spain), and 20 trained volunteers and two coordinators collaborated on this research. Participants were individually tested in a quiet room outside the classroom. Each subject was told that the experimenter was going to show him some games. All testing took place in the same session without breaks, and children were initially instructed on the various tasks and questionnaires. The child, seated at a table facing the computer, was presented with the materials, and the task was always presented in the same order: Deusto-e-motion1.0 and then the Feel test [27,30]. After the individual explanation, they completed the task during thirty minutes under standardized conditions within the school setting.

The ability to recognize facial expressions of the six basic emotions was investigated by using virtual faces in Deusto-e-motion1.0. Facial stimuli were presented to the subjects in 4 different blocks in the following order: (1) seven static facial emotions: neutral, happiness, anger, sadness, fear, surprise, and disgust; (2) six dynamic facial emotions, which included faces morphing from neutral to another emotion (neutral-happiness, neutral-anger, neutral-sadness, neutral-fear, neutral-surprise, and neutral-disgust); (3) four dynamic facial emotions which show faces morphing from one emotion to another: neutral-happiness-anger-sadness; and (4) seven static facial emotions: neutral, happiness, anger, sadness, fear, surprise, and disgust. The 24 virtual faces were shown one at a time, and the subjects were asked: “How is this person feeling?” They were asked to indicate the emotion depicted by the particular face as spontaneously as possible by choosing one button according to the following categories: happiness, anger, fear, sadness, disgust, surprise, or neutral. The order in which the blocks were presented to the subjects was the same in all the presentations. The duration of the stimuli was decided by a pilot study, which revealed that children needed about 3000 milliseconds to give a response. Emotional faces and labels were visible on screen at the same time. The program provided no feedback to the participants about the accuracy of their answers.

The second section of Deusto-e-motion1.0 included 30 items presenting social interactions and interpersonal conflicts within a school setting. Subjects had to decide the possible answer by choosing between the emotions happiness, anger, fear, sadness, disgust, surprise, or neutral. Social settings were illustrated by virtual animations that also incorporated recorded speech using a narrator’s voice. The first six items presented static pictures, whereas, in the rest of the task (24 items), the scenes were dynamic. The test questions all referred explicitly to a character’s feelings and the subject’s feelings. Answer options appeared in the right of the screen with the six emotions and the neutral option, and these were selected between by pressing the appropriate key. When the question had been read, the participant was required to press a specific button on the computer keyboard.

The Feel test [27] consisted of 42 photographs of actors and actresses showing static emotional faces (anger, fear, sadness, happiness, surprise, and disgust) on a computer screen for 300 milliseconds. Clicking on the appropriate box, subjects had to decide which emotion they had previously seen. Emotional pictures and labels were not visible on the screen at the same time. The Feel score took the accuracy and reaction time of the answers into consideration and ranged between 0 to 84 points.

Participation in the study was voluntary, confidentiality was ensured, and all the requirements established by the bioethical commission for studies with human beings were met.

### Statistical Analyses

Descriptive analyses were performed to assess the socio-demographic and clinical characteristics of the respondents. A Kolmogorov-Smirnov test was applied to evaluate the normal distribution of variables. The analysis showed that all variables were nonnormal. A Mann-Whitney *U* test and Kruskal Wallis test were used to investigate differences regarding age and gender for continuous variables. The chi-squared test was applied to the categorical variables and the Spearman test to the correlations. SPSS statistical package version 15 (IBM Corporation, Armonk, New York, United States) was used to analyze the data. Any *P*<0.05 was considered to be statistically significant.

## Results

### Content Validity and Piloting

A team of five psychologists, psychopedagogues, and computer scientists was involved in the design phase, specifically in generating ideas, characters, scenes, and instructions through brainstorming. The interjudge agreement was assessed with Kappa calculations (*k*=0.85). The values were within the range of fair to good agreement. The created virtual facial expressions were validated in a pilot study. For this purpose, 30 volunteers evaluated the facial material (48 faces) according to the expressed emotion. A final set of 24 items were chosen. After face and content validation, the tool was piloted. A total of 100 children were asked for their overall impression of the software, and whether any items had been challenging to answer. Following the pilot phase, the wording of item number 25 was modified slightly to prevent misunderstanding, and a section was added from the previous version because of the improvement of the static fear face. After this modification, the game was clear and understandable.

### Internal Validity of E-motion1.0

The internal validity of the instrument was examined using the Spearman correlation. The total score correlated positively with the static facial emotions’ score (r^2^=0.812; *P*<.001) and with the dynamic facial emotions’ score (r^2^=0.872; *P*<.001). Static facial emotions’ score correlated with dynamic facial emotions’ score (r^2^=0.424; *P*<.001). The reaction time scores of static faces correlated positively with the reaction time scores of dynamic faces (r^2^=0.706; *P*<.001).

### The Concurrent Validity of E-motion1.0

Concurrent validity compares scores of an instrument with the current performance of some other measure. In this study, it was determined through correlation analysis (Spearman rank-order correlation) of the first section of Deusto-e-motion1.0 [30], specifically the section which includes the facial recognition task, and the Feel test [27,30]. The correlation coefficient between the facial recognition total scores of Deusto-e-motion1.0 and those of the Feel was r^2^=0.339 (*P*<.001). The correlation coefficient between the facial recognition`s reaction time scores of Deusto-e-motion1.0 and those of the Feel was r^2^=0.508 (*P*<.001). The results showed small to moderate significant correlations between all Deusto-e-motion1.0 scales and the Feel scales in total scores and reaction time scores (Table 3).

**Table 3:** Spearman's Rho correlations between Emotion and Feel test (N=1236).

Feel test expressions			Deusto-e-motion1.0 expressions											
			Happiness		Surprise		Anger		Fear		Sadness		Disgust	
			C^a^	RT^b^	C	RT	C	RT	C	RT	C	RT	C	RT
**Happiness**			—^c^	—	—	—	—	—	—	—	—	—	—	—
	**C**		0.180	—	—	—	—	—	—	—	—	—	—	—
		*P* value	.02	—	—	—	—	—	—	—	—	—	—	—
	**RT**		—	0.448	—	—	—	—	—	—	—	—	—	—
		*P* value	—	.01	—	—	—	—	—	—	—	—	—	—
**Surprise**			—	—	—	—	—	—	—	—	—	—	—	—
	**C**		—	—	0.274	—	—	—	—	—	—	—	—	—
		*P* value	—	—	.03	—	—	—	—	—	—	—	—	—
	**RT**		—	—	—	0.394	—	—	—	—	—	—	—	—
		*P* value	—	—	—	.01	—	—	—	—	—	—	—	—
**Anger**			—	—	—	—	—	—	—	—	—	—	—	—
	**C**		—	—	—	—	0.127	—	—	—	—	—	—	—
		*P* value	—	—	—	—	.02	—	—	—	—	—	—	—
	**RT**		—	—	—	—	—	0.368	—	—	—	—	—	—
		*P* value	—	—	—	—	—	.01	—	—	—	—	—	—
**Fear**			—	—	—	—	—	—	—	—	—	—	—	—
	**C**		—	—	—	—	—	—	0.191	—	—	—	—	—
		*P* value	—	—	—	—	—	—	.26	—	—	—	—	—
	**RT**		—	—	—	—	—	—	—	0.215	—	—	—	—
		*P* value	—	—	—	—	—	—	—	.05	—	—	—	—
**Sadness**			—	—	—	—	—	—	—	—	—	—	—	—
	**C**		—	—	—	—	—	—	—	—	0.227	—	—	—
		*P* value	—	—	—	—	—	—	—	—	.01	—	—	—
	**RT**		—	—	—	—	—	—	—	—	—	0.375	—	—
		*P* value	—	—	—	—	—	—	—	—	—	.03	—	—
**Disgust**			—	—	—	—	—	—	—	—	—	—	—	—
	**C**		—	—	—	—	—	—	—	—	—	—	0.105	—
		*P* value	—	—	—	—	—	—	—	—	—	—	.04	—
	**RT**		—	—	—	—	—	—	—	—	—	—	—	0.292
		*P* value	—	—	—	—	—	—	—	—	—	—	—	.04

^a^C: correct.

^b^RT: reaction time.

^c^Not applicable.

### Discriminant Validity

#### Effect of Age and Gender on Facial Recognition

A Mann-Whitney *U* test was used to compare genders. Overall, there were no significant differences except for static score (*z*=–2.12; *P*=.03), dynamic score (*z*=–2.32; *P*=.02), sadness score (*z*=–2.10; *P*=.04), and disgust score (*z*=–2.85; *P*=.004). The size effect was low (0.1) in all the situations.

A Kruskal-Wallis test was conducted to investigate age differences. There were significant differences in static score (*X*^2^_13_=20.9; *P*<.001), dynamic score (*X*^2^_10_=18.99; *P*<.001), neutral score (*X*^2^_2_=18.99; *P*<.001), disgust score (*X*^2^_2_=29.46; *P*<.001), surprise score (*X*^2^_2_=29.46; *P*<.001), and all of the reaction time scores (*P*<.001). Results showed that the older the participants, the higher the total score, and the shorter the reaction time.

#### Effect of Age and Gender on Virtual Scene Answers

Comparisons of the answers and reaction times’ scores concerning gender and age were made using a Mann-Whitney *U* test (reaction time and gender), a Kruskal-Wallis test (reaction time and age) for a continuous variable, and the chi-squared test for categorical variables (type of answer; gender and type of answer and age). Overall, there were no significant differences in gender. However, age is an important variable to compare both total answer scores and reaction time scores (Tables 4 and 5). As in the facial recognition task, results showed that the older the participants, the slower their reaction time.

### Normative Data

The percentiles for the main scales of Deusto-e-motion were calculated, as shown in Table 6.

**Table 4:** Answer scores in virtual scenes regarding gender and age (N=1236).

Virtual scene item	Gender/answer		Age/answer	
	*X*^2^ (df^a^)	*P* value	*X*^2^ (df)	*P* value
8.1	—^b^	—	39.38 (21)	.009
8.2	—	—	—	—
9.1	—	—	33.61 (21)	.04
9.2	—	—	37.08 (21)	.02
10.1	—	—	58.95 (21)	<.001
10.2	—	—	46.79 (21)	.001
12	—	—	36.87 (21)	.02
14.1	—	—	—	—
14.2	—	—	41.65 (21)	.005
14.3	—	—	—	—
15.1	—	—	—	—
15.2	—	—	34.47 (21)	.03
15.3	—	—	—	—
16	—	—	36.00 (21)	.02
17	—	—	104.40 (24)	<.001
18	—	—	80.09 (21)	<.001
19.1	—	—	33.21 (21)	.04
19.2	14.31 (7)	.05	36.12 (21)	.02
20.1	—	—	34.75 (21)	.30
20.2	17.56 (7)	.01	—	—
22.1	—	—	—	—
22.2	—	—	—	—
23.1	—	—	—	—
23.2	—	—	35.20 (21)	.03
24.1	—	—	—	—
24.2	—	—	—	—
25	—	—	—	—
26	—	—	69.47 (21)	<.001
27	18.71 (7)	.009	101.02 (21)	<.001

^a^df: degrees of freedom.

^b^Not applicable.

**Table 5:** Reaction time scores in virtual scenes regarding gender and age (N=1236).

Virtual scene item	Gender/reaction time		Age/reaction time	
	*U*	*P* value	H (df^a^)	*P* value
8.1	110,482	<.001	55.46 (3)	<.001
8.2	115,430	.003	35.81 (3)	<.001
9.1	—^b^	—	21.15 (3)	<.001
9.2	—	—	12.86 (3)	.005
10.1	115,506	.003	10.77 (3)	.01
10.2	—	—	42.80 (3)	<.001
12	118,489	.03	56.01 (3)	<.001
14.1	—	—	57.87 (3)	<.001
14.2	—	—	10.72 (3)	.01
14.3	—	—	56.28 (3)	<.001
15.1	117,667	.02	25.23 (3)	<.001
15.2	—	—	38.76 (3)	<.001
15.3	—	—	36.21 (3)	<.001
16	114,167	.002	9.79 (3)	.03
17	115,247	.004	16.04 (3)	.001
18	114,636	.003	—	—
19.1	117,550	.02	70.05 (3)	<.001
19.2	—	—	38.10 (3)	<.001
20.1	117,101	.02	24.88 (3)	<.001
20.2	111,511	<.001	12.97 (3)	<.001
22.1	110,845	<.001	50.09 (3)	<.001
22.2	111,859	<.001	48.27 (3)	<.001
23.1	—	—	35.05 (3)	<.001
23.2	—	—	—	<.001
24.1	112,095	<.001	10.50 (3)	.015
24.2	—	—	17.40 (3)	.001
25	—	—	—	—
26	114,896	<.001	49.32 (3)	<.001
27	109,969	.003	30.41 (3)	<.001

^a^df: degrees of freedom.

^b^Not applicable.

**Table 6:** Percentiles for the dimensions.

Percentiles	Total (ms)	Dynamics (ms)	Statics (ms)	RT^a^ total (ms)	RT statics (ms)	RT dynamics (ms)
1	3.0000	2.00	1.00	2651.52	2071.64	2139.46
10	10.00	6.00	3.00	3374.97	3245.94	3204.25
15	10.50	7.00	3.00	3604.79	3491.47	3369.97
20	11.00	7.00	4.00	3867.94	3709.60	3553.80
25	12.00	7.00	4.00	4144.01	3996.21	3777.55
30	12.00	8.00	5.00	4256.58	4248.02	3972.95
35	13.00	8.00	5.00	4464.35	4642.24	4251.47
40	13.00	8.00	5.00	4699.64	4838.28	4569.60
45	13.00	8.00	5.00	4919.47	5017.41	4719.77
50	14.00	8.00	5.00	5095.82	5356.14	4864.15
55	14.00	9.00	6.00	5521.94	5629.10	5122.80
60	14.00	9.00	6.00	5752.88	5956.14	5333.10
65	15.00	9.00	6.00	5990.67	6332.60	5706.20
70	15.00	9.00	6.00	6210.44	6623.57	6002.10
75	15.00	9.00	6.00	6612.55	6967.42	6536.02
80	15.60	9.00	6.00	6972.70	7407.71	6954.30
90	16.00	10.00	7.00	8356.32	9064.51	8497.85
95	17.00	10.00	7.00	9810.77	10,379.68	10,230.6
99	17.00	10.00	7.00	12,219.42	13,517.41	14,087.2

^a^RT: reaction time.

## Discussion

According to Salovey [32], the skills associated with emotional intelligence include the assessment, expression, and regulation of one’s own emotions as well as those of others, and the understanding of emotions and their use in an adaptive way to perform other activities, such as cognitive or behavioral tasks. In this context, the face is the way that emotions can be exteriorized and expressed in a nonverbal way, something essential for children to adapt to their social environment [33].

Deusto-e-motion1.0 is a serious game designed to evaluate components of the theory of mind, specifically facial recognition and empathy in 8-11-year-old children. This study presents the design and validation of the Spanish version of the Deusto-e-motion1.0 serious game.

There is no doubt that a test with suitable psychometric properties contrasted with a representative sample of participants would be beneficial for both the evaluation of the ability to recognize emotional facial expressions and for planning individual or group intervention programs in the area of children’s interpersonal relationships. Moreover, the importance of developing such instruments with demonstrated validity and reliability to have appropriate protocols and paradigms to be applied in basic research should be highlighted, as in the case of neuroimaging studies [34].

This article explores content validity and piloting, internal validity, concurrent validity, and discriminant validity. The test shows moderate concurrent validity by correlating its scores with the Feel test that assesses similar capacity for emotion recognition. These results may be mediated by the characteristics of each test, in the sense that the Deusto-e-motion1.0 test includes, on the one hand, fewer items, and on the other, items with a dynamic and static nature. When working with variables of a very diverse nature, like different types of facial expression stimuli (cultural precedence or faces, photographs versus drawings), a lower correlation will support a high relation. It is suggested that in future studies, the rate of concurrent validity is calculated with a questionnaire with a higher number of static items [35].

There were significant differences in each gender’s scores for the static, dynamic, sadness, and disgust emotions. Results showed that the younger the age of the participants, the slower the reaction time. Overall, there were no significant differences in each gender’s scores in the virtual scenes section. However, age is an important variable to compare both the total answer scores and reaction time scores. With increasing age, facial expression recognition becomes faster and more accurate, possibly due to increased efficiency in understanding faces [36,37]. It is generally accepted that children’s ability to recognize the emotions of unfamiliar faces improves between the ages of 5-10 years old [38].

It should be noted that this study is not without its limitations, and results should be considered with caution. First, the results only indicate the comparability of the classic basic emotions, as described by Ekman. However, daily, pure basic emotions are encountered only rarely. Future research should primarily focus on investigating more ambiguous and nuanced emotional expressions. Second, the Feel test presents only static pictures of adults of Asian and European descent, whereas Deusto-e-motion1.0 presents static and dynamic virtual faces of a boy. Third, the test shows a higher percentage of masculine faces. In the development of a new version of the instrument, stimuli of female faces will be included.

## Abbreviations

EMMA: Engaging Media for Mental Health Applications
FEFA: Frankfurt Test and Training of Social Affect
VEPSY: updated telemedicine and portable virtual environments for clinical psychology
